# Effect of High-Voltage Additives on Formation of Solid Electrolyte Interphases in Lithium-Ion Batteries

**DOI:** 10.3390/ma15103662

**Published:** 2022-05-20

**Authors:** Minjing Chen, Yunbo Huang, Zhepu Shi, Hao Luo, Zhaoping Liu, Cai Shen

**Affiliations:** 1 Ningbo Institute of Materials Technology & Engineering Chinese Academy of Sciences, 1219 Zhongguan Road, Zhenhai District, Ningbo 315201, China; chenminjing@nimte.ac.cn (M.C.); 15605219893@163.com (Y.H.); shizhepu@nimte.ac.cn (Z.S.); luoh@nimte.ac.cn (H.L.); 2 Nano Science and Technology Institute, University of Science and Technology of China, 166 Renai Road, Suzhou Industrial Park, Suzhou 215123, China; 3 China Beacons Institute, University of Nottingham Ningbo China, 211 Xingguang Road, Ningbo 315100, China

**Keywords:** solid electrolyte interphase, high-voltage electrolyte, additives, electrochemical atomic force microscopy, lithium-ion batteries

## Abstract

Solid electrolyte interphase (SEI) formed at the interface in lithium-ion batteries plays an important role in isolating electrons and permeating ions during charging/discharging processes. Therefore, the formation of a good interface is crucial for better battery performance. In this study, additives based on adiponitrile (ADN) and trimethyl borate (TMB) were employed to broaden the electrochemical window and form a good SEI layer. Electrochemical Atomic force microscopy (EC-AFM) was used for in situ studies of film-formation mechanisms in high-voltage electrolytes on high-temperature pyrolytic graphite (HOPG), as well as Li- and Mn-rich (LMR) materials. X-ray photoelectron spectroscopy (XPS) combined with electrochemical methods revealed a synergistic reaction between the two additives to form a more stable interfacial film during charging/discharging processes to yield assembled batteries with improved cycle performance, its capacity increased from below 100 mAh/g to 200 mAh/g after 50 cycles. In sum, these findings would have great significance for the development of high voltage lithium-ion batteries with enhanced performance.

## 1. Introduction

The increase in global warming and other environmental issues has encouraged more focus on the development of new electric vehicles. The main power source of electric vehicles is currently based on lithium-ion batteries (LIBs), making the development of high-energy-density LIBs important [1,2]. As secondary batteries, LIBs are advantageous in terms of their light weight, high energy density, good memory effect, and long cycle life [3,4]. During the first few cycles of charge/discharge processes of LIBs, solid electrolyte interphase (SEI) film formed on the electrodes helps the transport of lithium ions and restricts electron tunneling, thereby preventing the detrimental oxidative decomposition of the electrolyte [5,6,7]. The formation of uniform and intact SEI films may effectively improve the cycling performance and safety of LIBs. By contrast, poorly formed interface films can form lithium dendrites, resulting in high interfacial impedance and capacity fading, resulting in safety issues in the LIBs [8,9]. In recent years, some researchers have constructed artificial SEI films by chemical vapor deposition (CVD) [10,11], which provide a fast migration channel for lithium ions and improve the cycle performance of batteries, but this method often requires high costs.

The key to developing high-energy-density LIBs lies in finding suitable high-voltage cathode materials. Li- and Mn-rich (LMR) materials possess high energy density (>350 mAh/g) and an elevated discharge platform, and are thereby considered promising cathode materials for next-generation LIBs. Common carbonate electrolytes generally consist of lithium hexafluorophosphate (LiPF_6_), lithium difluoro(oxalate)borate (LiDFOB), bis(trifluoromethanesulfonyl)imide (LiTFSI), or lithium tetrafluoroborate (LiBF_4_) as lithium salts, as well as ethyl carbonate (EC), dimethyl carbonate (DMC), diethyl carbonate (DEC), or methyl ethyl carbonate (EMC) as electrolyte solvents. However, such carbonates may undergo severe oxidation at 4.3 V or even at lower potentials, leading to the generation of interfacial gas, low Coulombic efficiency, and poor capacity retention rate [12].

There are two main methods for solving the above issues, the first is to find new types of high-voltage electrolyte. Alvarado et al. discovered a carbonate-free electrolyte (3.25 M LiTFSI SL) with an electrochemical window as high as 5.5 V; the discharge capacity of the assembled battery could be maintained at 69% after long cycle test, compared to the quick fade to 0 mAh/g of the battery assembled using the carbonate electrolyte (1.2 M LiPF_6_ EC/EMC) [13]. Tornheim et al. discovered a highly fluorinated electrolyte (1.2 M LiPF_6_ HFDEC) that maintained a capacity of 189 mAh/g after 100 cycles on a battery containing graphite anodes, compared to 165 mAh/g for the battery assembled with the standard electrolyte (1.2 M LiPF_6_ EC/EMC) [14]. The second way is to add a small amount of functional high-voltage additives to the electrolyte; this method is more cost-effective and environmentally friendly. Functional high-voltage additives are often mixed with the electrolyte to form a stable interfacial film to improve the battery cycle performance [15]. New types of high-voltage electrolyte additives include boron-containing additives, nitrile-containing additives, and fluorine-containing additives. Li et al. added 2% trimethyl borate (TMB) to common carbonate electrolyte and noticed improved stability of interfacial film by oxidation and reduction at the ternary single crystal cathode interface before other solvent components, inhibiting the self-discharge of the battery [16]. Rong et al. added 1% Tris (trimethylsilyl) borate (TMSB) and recorded a remaining capacity rate of 95.3% in the (LNMO||Li) battery after 200 cycles at a rate of 0.5 C. Scanning electron microscopy (SEM) and transmission electron microscopy (TEM) revealed the formation of uniform and thin interface film at the cathode interface, effectively inhibiting the continuous oxidation and decomposition of the electrolyte, as well as preventing the dissolution of transition metals Mn and Ni [17]. In general, nitrile additives have high electrochemical windows, high anode stabilities, low viscosities, and elevated boiling points. As a result, Wang et al. found that the addition of adiponitrile (ADN) could reduce the interface impedance and form a stable interfacial film, thereby reducing the dissolution of cathode particles at the interface and improving the battery performance [18]. Traditional additives such as VC and FEC are good anode film formers, but VC shows a weak antioxidant capability and is unstable at high voltages in cathode materials [19]. FEC is regarded as being beneficial due to its good film-forming properties on graphite anodes; it is not suitable for high-voltage lithium-ion batteries because it facilitates Li metal dendrite penetration and aggravates the respective issues associated with rollover failure [20].

The boron atom in the additive TMB has an affinity for electrons; it can combine with the anion in the electrolyte and stabilize the carbonate electrolyte. In addition, TMB has excellent cathode film-forming properties, so it is considered as an additive in this research. Although ADN is considered to be a hazardous chemical, it has a high electrochemical window of up to 6.9 V and high stability, and has been widely used to improve the cycle stability of high-voltage cathode materials. The strong complexation of nitrile can inhibit the oxidation reaction of the electrolyte on the surface of high-voltage cathode materials and remove the hydrofluoric acid generated by the hydrolysis of trace water and lithium salt in the electrolyte [21]. Based on these advantages, ADN was added to the electrolyte as an additive in small amounts. In recent years, LiDFOB has been reported as an additive for forming the passivation layer on both electrodes and reducing the capacity loss of assembled batteries [22]. However, in our research, LiDFOB was picked as the lithium salt due to its stability for film-formation and high solubility in electrolyte solvents [23]. Dimethyl sulfite (DMS) was employed as a high-voltage solvent component, and both TMB and ADN were synergistically added as additives to broaden the electrochemical window and explore how both additives affect high-voltage electrolytes.

Currently used methods for observing solid electrolyte interface films include X-ray photoelectron spectroscopy (XPS) [24,25], nuclear magnetic resonance (NMR) [26,27], electrochemical impedance spectroscopy (EIS) [28,29], vacuum transmission electron microscopy (TEM) [30,31], and scanning electron microscopy (SEM) [32,33]. In the present article, atomic force microscopy (AFM) [34] was employed owing to its ability to detect the in situ dynamic growth of SEI films. The observation of cathode electrolyte interphase (CEI) films is often more challenging than that of SEI films, owing to the relatively rough and uneven aspects of the cathode particles [35,36], not to mention the difficulty of observation under fluid conditions. Recent research dealing with interfacial cathode materials has focused on lithium-ion migration behavior on the particle surface, explaining why lithium ions tend to move along the grain boundaries [37,38,39]. However, such research has not yet uncovered the morphological evolution of the cathode electrolyte interphase (CEI). Therefore, the present article is mainly devoted to the application of high-voltage electrolytes on highly oriented pyrolytic graphite (HOPG) anode based on predecessors [40,41]. The application of optimized electrolytes in high-energy-density cathodes based on LMR materials is also investigated in situ [42,43,44].

## 2. Experimental Section

### 2.1. Methods

The electrode used as an anode was made of a smooth and flat HOPG (Bruker Corporation, Billerica, MA, USA, 12 × 12 × 2 mm). The cathode electrode was composed of Li- and Mn-rich (LMR) material Li_1.14_Ni_0.13_Co_0.13_Mn_0.54_O_2_ as active material mixed with super-p and N-Methyl pyrrolidone (8:1:1, mt%) to form a mixture, which was stirred in ball grinder for 2 h then applied to Al foil followed by vacuum drying at 120 °C for at least 24 h. All electrolytes were prepared in the glove box (MBRAUN, argon-filled, O_2_ ≤ 0.1 ppm, H_2_O ≤ 0.1 ppm). The Base 1 electrolyte (B1) consisted of 0.7 M/L lithium difluoro(oxalate)borate (LiDFOB) (Sigma-Aldrich, Shanghai, China) and ethyl carbonate (EC) (Sigma-Aldrich, Shanghai, China) as solvent. The Base 2 electrolyte (B2) was obtained by adding dimethyl sulfite (DMS) (Sigma-Aldrich, Shanghai, China) at a volume ratio of DMS:EC of 7:3. Electrolyte 1 and 2 (E1 and E2) were prepared by adding additives 4 vt% trimethyl borate (TMB) (Sigma-Aldrich, Shanghai, China) and 2 vt% adiponitrile (ADN) (Sigma-Aldrich, Shanghai, China), respectively. Electrolyte 3 (E3) was prepared by adding both additives as the final optimized high-voltage electrolyte. The prepared electrolytes are listed in Table 1.

### 2.2. AFM Measurements

In situ EC-AFM experiments were carried out in the glove box. The device was made of an electrochemical workstation and an atomic force microscope (Icon, Bruker Corporation, Billerica, MA, USA). Lithium bars were used as the reference electrode (RE) and counter electrode (CE), and copper wire was connected to the electrode piece as a working electrode (WE). One end of the device was connected to AFM, and the other end was connected to the electrochemical workstation (CH760, Shanghai Chenghua Corporation, Shanghai, China). Under the applied voltage, the device was used for continuous in situ observation with AFM. The SCANASYST-FLUID+ probe was employed for EC-AFM tests at a scan rate of 1 Hz per line and an image resolution of 256 × 256 pixels.

### 2.3. Materials Characterization

The batteries after three charge/discharge processes were first disassembled and the samples were taken out and rinsed several times with dimethyl carbonate (DMC) to remove the solvent. X-ray photoelectron spectroscopy (XPS, AXIS ULTRA DLD, Kratos, Manchester, UK) was used to analyze the chemical structure of each sample surface.

### 2.4. Electrochemical Testing

The electrochemical test samples were all made of 2032-type coin cells. The electrochemical window of the electrolyte was measured by the electrochemical workstation using linear scanning voltammetry (LSV). The scanning voltage ranged from 3 V to 7 V at a scan rate of 0.5 mV/s. Cyclic voltammetry (CV) was carried out at voltages from 3 V to 0 V on the electrochemical station for (C||Li) battery and from 2 V to 4.8 V for (LMR||Li) battery at a scanning speed of 0.5 mV/s. The electrochemical impedance spectroscopy (EIS) tests were recorded on the electrochemical workstation. The samples were made of coin cells (C||Li and LMR||Li) that required standing still for at least 2 h before testing. The frequency range for AC impedance varied from 0.01 Hz to 1 MHz and the disturbance voltage was 10 mV. The discharge capacity and Coulombic efficiency of each battery were measured by LAND battery test system (BT2013A, Wuhan Lanbo Test Equipment Co., Ltd., Wuhan, China). The voltage range of the graphite anode was set to be 3–0 V, and the measurement voltage range of LMR cathode was 2–4.8 V. The cycling speeds for the first three cycles were set to be 0.1 C and 0.2 C for subsequent cycles.

### 2.5. Gas Generation Behavior

Differential electrochemical mass spectrometry (DEMS) was applied to test the gas-generated components in the electrolyte during CV cycling. To this end, the half-cells were assembled in the mold inside of the glovebox. The sample was then connected to the carrier gas path, and a U-shaped tube was placed inside with added dry ice. The connection of metal to the glass interface and pipe from the steel cylinder to the flow meter opened the cylinder main valve and pressure reducing valve, as well as set up the flow meter. The connection of the pipeline to the battery unplugged a special wire and inserted the gas wire. Before starting, bubble water was used to check for any leaks in the connection port. Afterward, the flow rate of the flow meter was set, and the mass spectroscopy baseline was opened. Cyclic voltammetry tests were next set up on the connected electrochemical workstation at the scanning range from 2 V to 4.8 V and scanning rate of 0.5 mV/s. A total of three cycles were performed, and the detected gas was identified as carbon dioxide (CO_2_).

## 3. Results and Discussion

In this research, the electrochemical windows of various electrolytes were first investigated by LSV tests. In situ EC-AFM tests on the HOPG of different electrolytes were applied for studying the dynamic evolution of the interfacial films. The electrolyte E3 was further applied to the surface of the high-voltage LMR material for in situ EC-AFM tests. DEMS tests were performed to study the gas production behavior on the LMR surface. EIS testing and XPS analysis were applied to characterize the change and composition of the solid electrolyte films. In the end, the batteries were assembled to test the effect of different electrolytes on the cycle performance of the batteries.

### 3.1. LSV Tests

**Figure 1:**
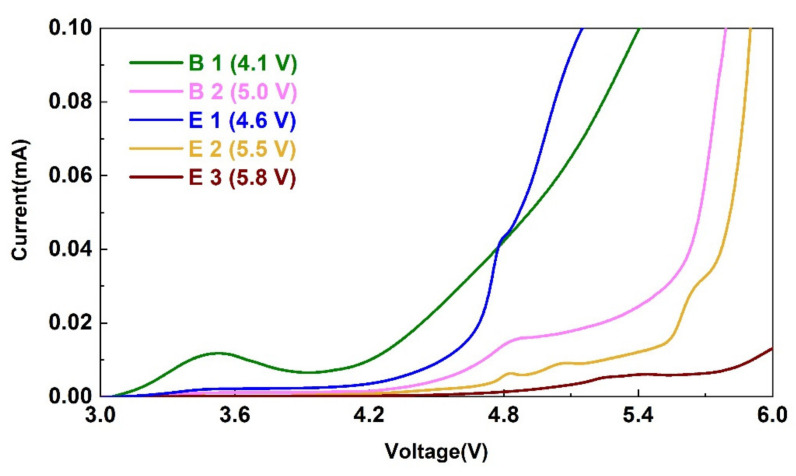
Linear scanning voltammetry curves in different electrolytes.

The electrochemical stability of each electrolyte is shown in Figure 1. Using lithium salt LiDFOB, the electrolyte with only EC as solvent showed a huge oxidation peak around 4.1 V, indicating the start of the oxidation and decomposition of the electrolyte B1 at this potential. After adding the high-voltage component DMS, the electrochemical window of electrolyte B2 broadened to 5.0 V. After adding 2 vt% ADN, the electrochemical window of electrolyte E2 further increased to about 5.5 V, clearly showing its function in broadening the electrochemical window. However, the addition of 4 vt% TMB did not show significant improvement in the electrochemical window of the electrolyte E1 when compared to that of the electrolyte B2. However, the addition of the two additives obviously enhanced the electrochemical window of electrolyte E3 up to about 5.8 V. Hence, the electrolyte with synergic functions of the two additives showed the best oxidation resistance.

### 3.2. In Situ EC-AFM of Different Electrolytes on HOPG

**Figure 2:**
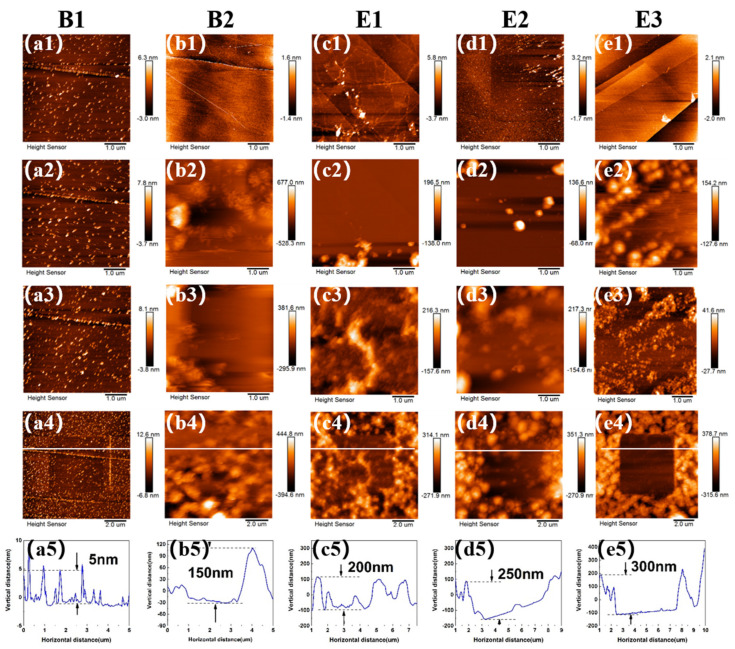
(**a**–**e**) In situ AFM images of HOPG electrodes in different electrolytes. (First row, **a1**–**e1**) HOPG surface before CV cycling, (second row, **a2**–**e2**) 2–2.5 V in 1st cycle, (third row, **a3**–**e3**) after three cycles of CV, (fourth row, **a4**–**e4**) zoomed−out images of the scratched area, (fifth row, **a5**–**e5**) line profile analysis of SEI film scratched.

To accurately observe the changes in the growth of interfacial films, EC-AFM tests were conducted in the selected electrolytes. Figure 2 presents the morphological changes of SEI film at HOPG surface in different electrolytes during CV cycling. After injection of the electrolyte B1, some small particles immediately appeared, as shown in Figure 2a1. When the voltage was swept from 3 V to 1.5 V, small particles emerged at the bottom of HOPG surface. Figure S1 shows the CV curves of different electrolytes on HOPG in EC-AFM tests from 3 V to 0 V. At around 0.9 V, at which point EC decomposes [40], more small-sized particles were noticed, which continued to grow and deposit (Figure 2a4). After three cycles, the thickness of the film was measured to be 5 nm (Figure 2a5).

The addition of the electrolyte B2 containing DMS as a high-voltage solvent component led to the formation of large particles, which started to deposit at 2.5 V (Figure 2b2). Please note that this voltage corresponds to the decomposition potential of DMS (Figure S1). The adhesion of particles during growth was not strong enough, and therefore, SEI film could easily be scraped off under the slight force of the probe. After three cycles, the thickness of the film reached 150 nm (Figure 2b5).

The addition of the electrolyte E1 containing the film-forming additive TMB led to the decomposition of particles at 2.25 V (Figure 2c2), yielding a relative uniform particle size, but the growth was not dense enough (Figure 2c4). This could be the explanation for the narrow electrochemical window in Figure 1. After three cycles, the thickness of the film reached 200 nm (Figure 2c5).

As shown in Figure 2d2, the addition of electrolyte E2 containing ADN to widen the electrochemical window led to the occurrence of a large amount of particle growth at 2.35 V, corresponding to the decomposition potential of ADN (Figure S1). Compared to the electrolyte without ADN, the particle morphology looked more different in size and particle growth was denser than in that containing TMB only. After three cycles, the thickness of the film increased to 250 nm (Figure 2d5).

The addition of the electrolyte E3 containing ADN and TMB led to the growth of large particles at around 2.2 V (Figure 2e2 and Figure S1). Please note that particles grew along the edge of the step initially, and then covered the whole HOPG surface. The resulting film was relatively dense after three cycles, with thickness increasing to 300 nm (Figure 2e5). Combined with the LSV data in Figure 1, it can be concluded that ADN played a role in broadening the electrochemical window, while the role of TMB was to form a dense film. In the presence of only TMB, the film formed was not dense enough to resist the oxidation of the electrolyte. Thus, both additives synergistically generated SEI film with a better antioxidant effect.

### 3.3. In Situ EC-AFM of E3 on LMR Cathode

**Figure 3:**
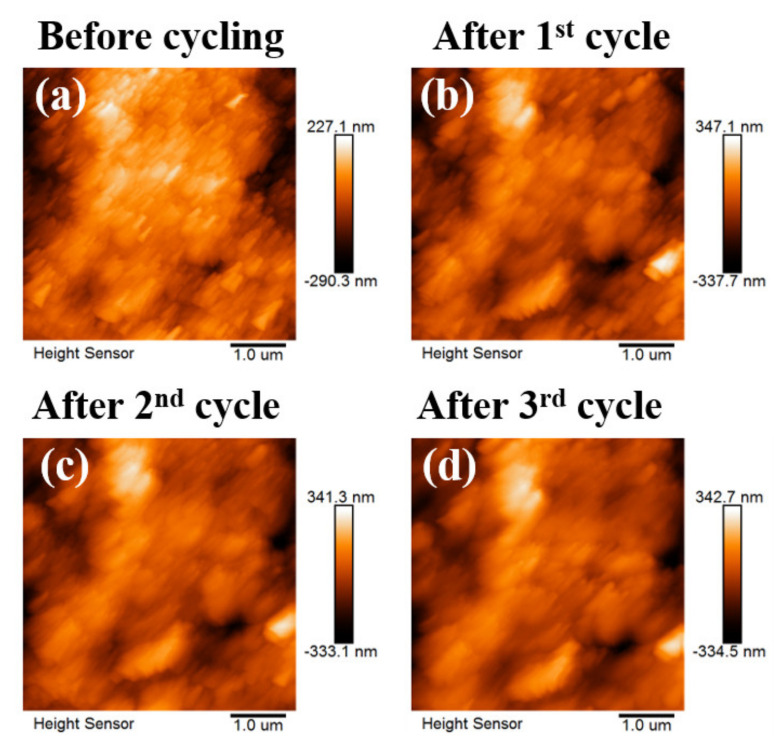
In situ AFM images of electrolyte E3 in LMR material (5 µm × 5 µm). (**a**) AFM images of LMR particles in air before cycling. (**b**–**d**) AFM images of LMR particles in the electrolyte after first, second, and third CV cycle.

The AFM images of CEI formation on LMR cathode during cycling are provided in Figure 3. LMR particles with round shapes can be observed in Figure S2a1. When the voltage was controlled at 2 V and electrolyte E3 was injected, a layer of substance rapidly covered the surface at 4.3 V (Figure S2a3). This voltage also corresponded to the value at which the large oxidation peak showed up. After three cycles, the in situ image revealed a uniform CEI film covering the surfaces of LMR particles (Figure 3d). This acted as a stabilizer for the structure of cathode material and an inhibitor for undesirable oxidative decomposition.

### 3.4. DEMS Tests

**Figure 4:**
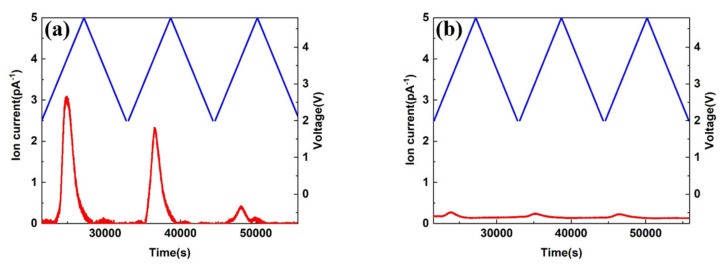
DEMS data of CO_2_ released in different electrolytes during cycling. The blue and red lines correspond to the vertical axes of voltage and ion current respectively. (**a**) 1 M LiPF_6_ EC/EMC/DMC and (**b**) E3.

Commonly used carbonate electrolytes oxidize in 4.3 V and cause severe gas formation. Hence, DEMS tests were carried out in high voltage electrolyte E3 and commonly used carbonate electrolyte (1 M LiPF_6_ EC/EMC/DMC) to clarify the differences in gas generation behavior on LMR cathode. As shown in Figure 4a, significant carbon dioxide (CO_2_) production peaks appeared during cycling from 2 V to 4.8 V. However, the CO_2_ peak produced on LMR cathode is relatively small in Figure 4b. The carbon dioxide originated from the oxidation of the electrolytes. Hence, high-voltage electrolytes could form stable passivation films on LMR cathode to prevent further oxidative decomposition of the electrolyte.

### 3.5. Electrochemical Impedance

**Figure 5:**
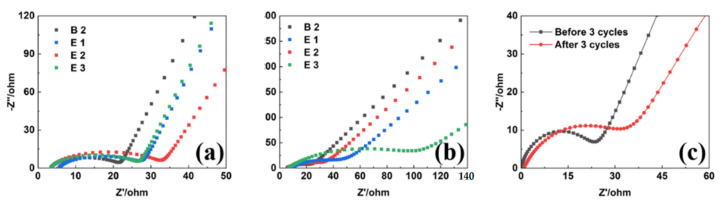
EIS analyses of C||Li batteries before (**a**) and after (**b**) 3 CV cycles. (**c**) EIS analysis of LMR||Li battery.

The interface film impedance data of the four electrolytes before and after three cycles in graphite anode and LMR cathode are illustrated in Figure 5. The high frequency and medium frequency regions represent the interface film impedance (R_SEI_) and charge diffusion impedance (R_ct_), respectively. Since EC remained solid at room temperature, its impedance value as a single solvent electrolyte far exceeded those of the other electrolytes (Figure S3). Additionally, this failed to form SEI film after three cycles, while the battery impedance of the electrolyte containing 4% TMB increased significantly, as can be seen in Figure 5b. The impedance of the battery without TMB was relatively reduced, and the resulting SEI film may not be dense enough or have sufficient ion permeability, confirming the role of TMB as a film-forming additive. After three cycles of activation on LMR cathode, the impedance value of the representative CEI film increased from 21.308 Ω to 31.318 Ω (Table 2), showing the formation of dense interface film after three cycles and increasing the interface impedance value.

### 3.6. XPS Analysis

**Figure 6:**
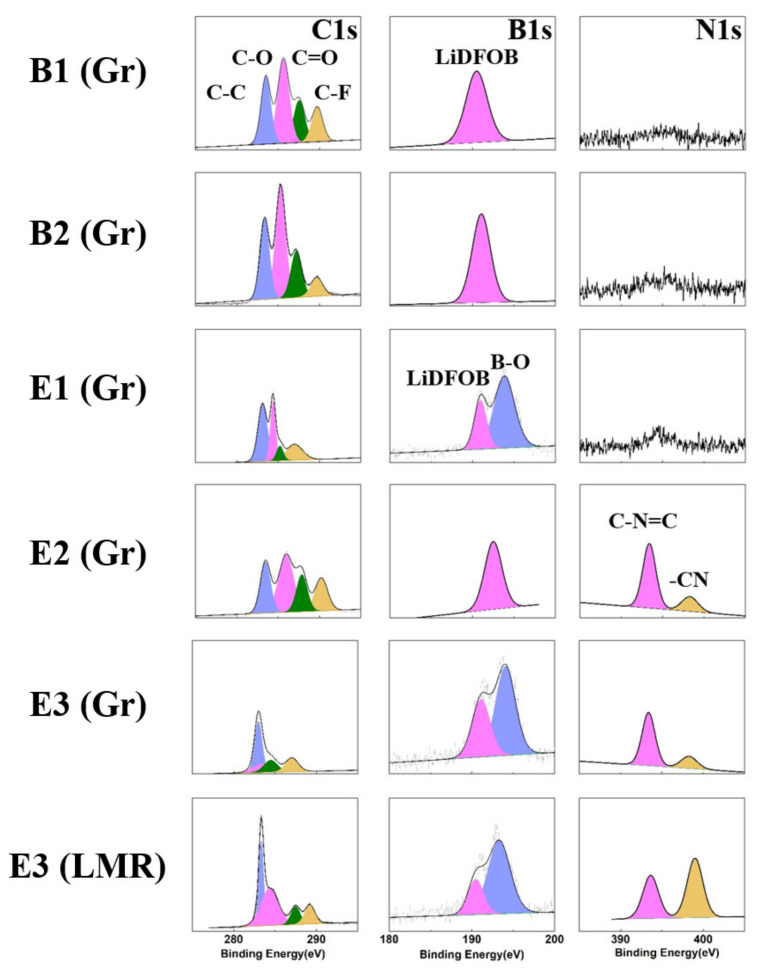
XPS analyses of different electrolytes on graphite anodes and LMR cathode.

Since AFM cannot provide detailed information on chemical compositions, XPS was used for composition characterization of the interface to better clarify the formation of the interface SEI film. Figure 6 shows the C1s, B1s, and N1s spectra of different electrolytes on graphite and LMR electrode. As shown in C1s spectra, typical vibration peaks of C-C and C-H bonds were noticed at 284.8 eV, representing the existence of Super P. The vibration peak of C-O-C bond at 286 eV and the component peak of carbonate at 289 eV corresponded to organic carbonates produced by the oxidative decomposition of the electrolyte. For the O1s spectra (Figures S4 and S5), the corresponding lithium carbonate and organic esters at 530 eV and 533 eV were produced by the oxidative decomposition of the electrolyte. Higher contents led to more serious electrolyte oxidative decomposition. From C1s in Figure 6 and Figure S5, the electrolyte containing the synergistic effect of both additives produced the lowest number of electrolyte products on the graphite surface when compared to the others.

In the F1s spectra of Figures S4 and S5, the binding energy at 690 eV represented the PVDF peak. A stronger peak induced thinner SEI film. In Figure S4, the PVDF peak remained strong in the electrolyte containing only EC as the solvent, confirming that this kind of electrolyte forms the thinnest films.

For the N1s and B1s spectra, the -CN and B-O bond peaks could be detected in the electrolyte containing ADN and TMB additives. Combined with AFM data analysis and previous research, it can be concluded that ADN formed a CN=C bond-containing film at the interface [18], while TMB formed a film containing particles with B-O bonds [16]. The particles in the interface film formed when using TMB as a film-forming additive were not dense enough, which explains the narrow electrochemical window of the electrolyte containing TMB as the only additive. This effect was more pronounced with the synergistic addition of ADN, where larger particles were generated and a denser interfacial film was formed. These features enhanced the stability of the interface and further inhibited the oxidative decomposition of the electrolyte.

### 3.7. Cycle Performance

**Figure 7:**
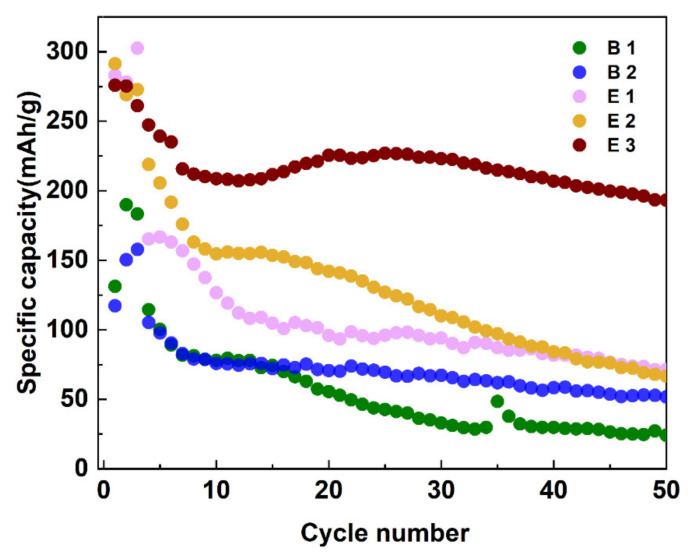
Cycle performances of different electrolytes in C||Li batteries at 0.1 C. The first three cycles were measured at a rate of 0.05 C for activation.

The cycle performance of the battery assembled with the electrolyte is shown in Figure 7. Here, the battery assembled with graphite cathode and lithium metal anode was tested using a LAND battery test system at voltages ranging from 2 V to 4.8 V and a scanning speed of 0.05 C for three activation cycles and then at 0.1 C for the following cycles. Here, specific capacity represents the specific discharge capacity, which was calculated by dividing the discharge capacity by the mass of the active materials. In Figure 7, the first specific capacity values of the batteries containing the additives were higher than those of the batteries containing electrolyte B1 and B2, confirming that both additives could work immediately after the addition. After 50 cycles, the specific capacity of the electrolyte E3 was maintained at about 200 mAh/g when compared to the battery containing electrolyte B1, showing that capacity decayed to below 100 mAh/g. The cycle performance of the assembled battery with the electrolyte E3 was obviously enhanced, indicating its usefulness in inhibiting serious oxidation and decomposition of the electrolyte, thereby delaying the capacity decay of the lithium battery.

## 4. Conclusions

A high-voltage electrolyte containing two additives was successfully used to form dense films and broaden the electrochemical window. The formed SEI film was relatively dense, and the assembled battery showed improved cycle performance. The application of high-voltage electrolyte to LMR materials led to the formation of a uniform CEI film on the cathode material. DEMS revealed less CO_2_ produced on the LMR materials due to the inhibited electrolyte oxidation by the CEI film formed from this electrolyte. In sum, these results clarify the formation of CEI of high-voltage electrolytes on popular LMR materials, and this electrolyte is useful in the development of high-energy-density lithium-ion batteries.

## Figures and Tables

**Table 1:** The prepared electrolytes used in the experiments.

Name	Electrolyte Components
Base 1 (B1)	0.7 M/L LiDFOB EC
Base 2 (B2)	0.7 M/L LiDFOB DMS/EC
Electrolyte 1 (E1)	0.7 M/L LiDFOB DMS/EC 4 vt% TMB
Electrolyte 2 (E2)	0.7 M/L LiDFOB DMS/EC 2 vt% ADN
Electrolyte 3 (E3)	0.7 M/L LiDFOB DMS/EC 4 vt% TMB 2 vt% ADN

**Table 2:** EIS data of graphite (Gr) anodes and LMR cathodes before (R_SEI_) and after (R_SEI’_) 3 CV cycles.

	R_SEI_/Ω	R_SEI’_/Ω
B2 (Gr)	21.764	19.112
E1 (Gr)	32.843	26.735
E2 (Gr)	27.814	46.79
E3 (Gr)	26.786	95.41
E3 (LMR)	21.308	31.318

## Data Availability

Not applicable.

## Supplementary Materials

The following supporting information can be downloaded at: https://www.mdpi.com/article/10.3390/ma15103662/s1, Figure S1: CV curves of in-situ AFM tests of five electrolytes on graphite from 3 V to 0 V; Figure S2: In-situ AFM images of the electrolyte E3 on the surface of LMR material. AFM images of 2 µm × 2 µm (a1–a4) during cycling and 5 µm × 5 µm (b1–b4) after each cycle; Figure S3: EIS analysis on graphite anode in the electrolyte B1; Figure S4: XPS analysis of different electrolytes on graphite anodes; Figure S5: XPS analysis of the electrolyte E3 on graphite anode and LMR cathode.
