# Supplementary material for: Effect of High-Voltage Additives on Formation of Solid Electrolyte Interphases in Lithium-Ion Batteries
Source: Materials (Basel). 2022 May 20;15(10):3662. doi: 10.3390/ma15103662 (PMC9144735; doi:10.3390/ma15103662)
Supplement: Supplementary file 1 [file materials-15-03662-s001.zip › materials-1658069-supplementary.pdf]

materials

MDPI

Effect of High-Voltage Additives on Formation of Solid Electrolyte Interphases in Lithium-Ion Batteries

Minjing Chen [1,2], Yunbo Huang [1], Zhepu Shi [1,3], Hao Luo [1], Zhaoping Liu [1,*] and Cai Shen [1,3,*]

1 Ningbo Institute of Materials Technology & Engineering Chinese Academy of Sciences, 1219 Zhongguan Road, Zhenhai District, Ningbo 315201, China; chenminjing@nimte.ac.cn (M.C.); 15605219893@163.com (Y.H.); shizhepu@nimte.ac.cn (Z.S.); luoh@nimte.ac.cn (H.L.)
2 Nano Science and Technology Institute, University of Science and Technology of China, 166 Renai Road, Suzhou Industrial Park, Suzhou 215123, China
3 China Beacons Institute, University of Nottingham Ningbo China, 211 Xingguang Road, Ningbo 315100, China
* Correspondence: liuzp@nimte.ac.cn (Z.L.); cai.shen@nottingham.edu.cn (C.S.)

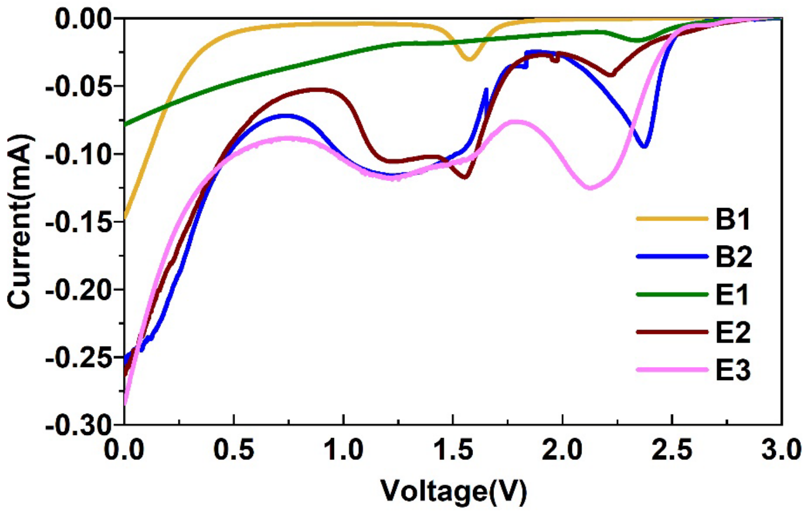

Figure S1. CV curves of in−situ AFM tests of five electrolytes on graphite from 3 V to 0 V.

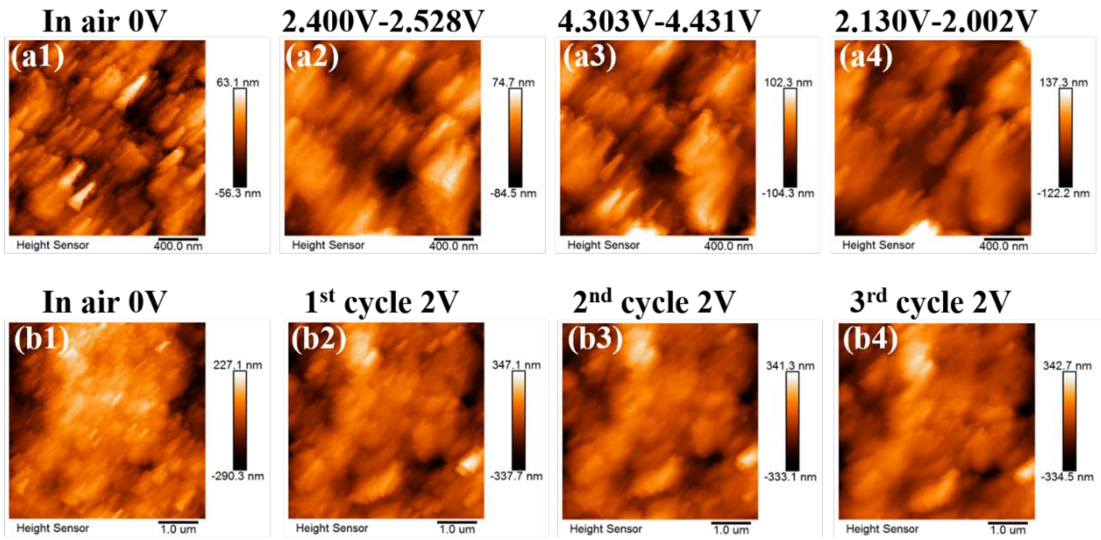

Figure S2. In-situ AFM images of the electrolyte E3 on the surface of LMR material. AFM images of 2 μm×2 μm (**a1**–**a4**) during cycling and 5 μm × 5 μm (**b1**–**b4**) after each cycle.

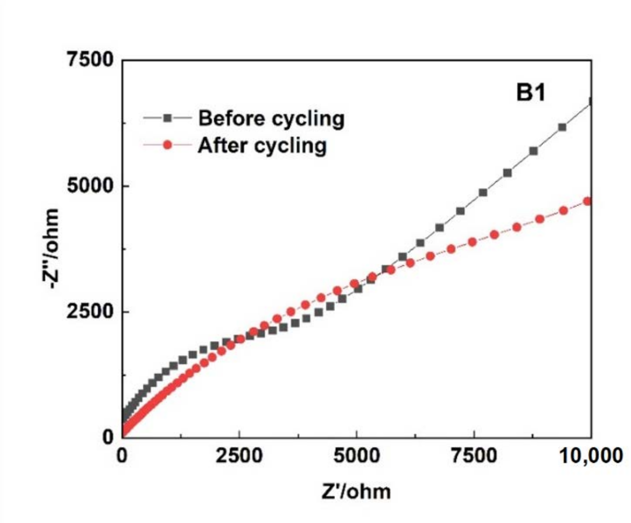

Figure S3. EIS analysis on graphite anode in the electrolyte B1.

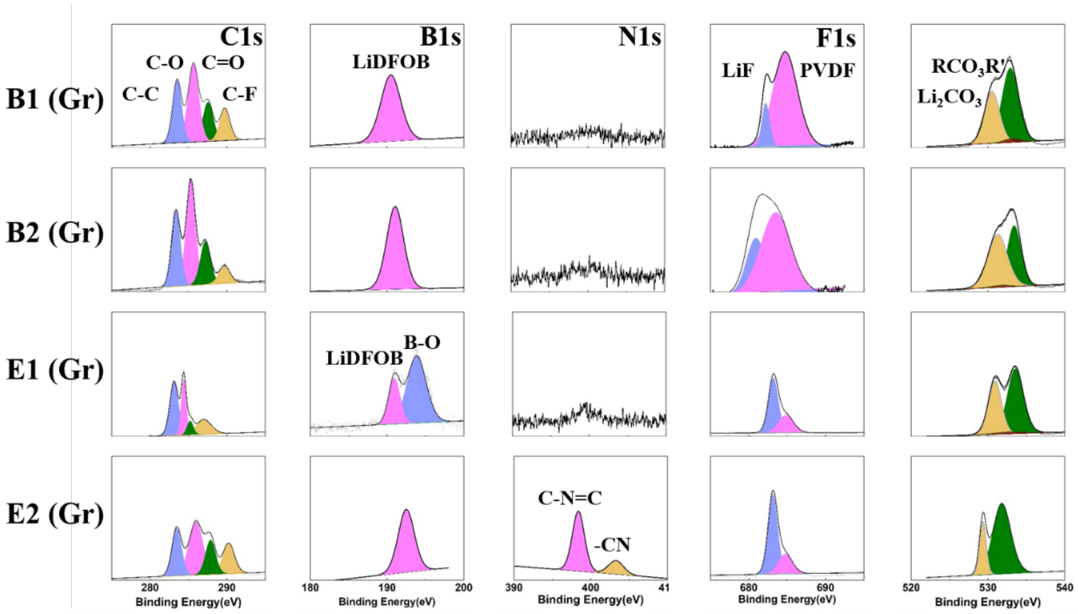

Figure S4. XPS analysis of different electrolytes on graphite anodes.

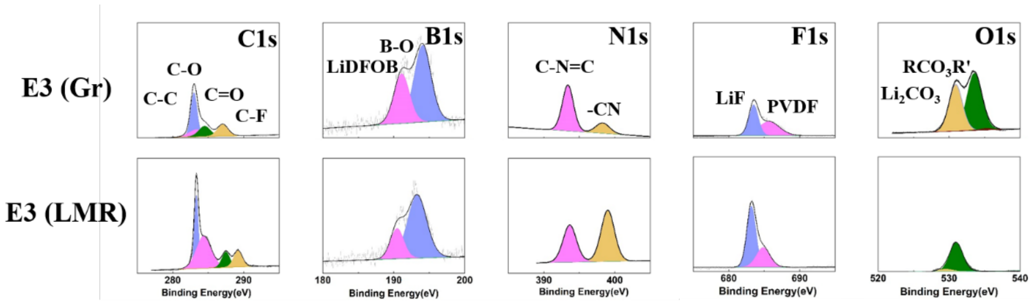

Figure S5. XPS analysis of the electrolyte E3 on graphite anode and LMR cathode.
